# Salt-responsive transcriptome analysis of triticale reveals candidate genes involved in the key metabolic pathway in response to salt stress

**DOI:** 10.1038/s41598-020-77686-8

**Published:** 2020-11-26

**Authors:** Chaohong Deng, Zhibin Zhang, Guorong Yan, Fan Wang, Lianjia Zhao, Ning Liu, Abudukeyoumu Abudurezike, Yushan Li, Wei Wang, Shubing Shi

**Affiliations:** 1 College of Agronomy, Xinjiang Agricultural University, Urumqi, 830091 China; 2 Research Institute of Crop Germplasm Resources, Xinjiang Academy of Agricultural Sciences, Urumqi, 830091 China; 3 State Key Laboratory of Cotton Biology, Institute of Cotton Research, Chinese Academy of Agricultural Sciences, Anyang, 455000 China; 4 Wulumuqi Sub-center for New Plant Variety Tests, Ministry of Agriculture and Rural Affairs, Urumqi, 830091 China

**Keywords:** Transcriptomics, Salt

## Abstract

Triticale is tolerant of many environmental stresses, especially highly resistant to salt stress. However, the molecular regulatory mechanism of triticale seedlings under salt stress conditions is still unclear so far. In this study, a salt-responsive transcriptome analysis was conducted to identify candidate genes or transcription factors related to salt tolerance in triticale. The root of salt-tolerant triticale cultivars TW004 with salt-treated and non-salt stress at different time points were sampled and subjected to de novo transcriptome sequencing. Total 877,858 uniquely assembled transcripts were identified and most contigs were annotated in public databases including nr, GO, KEGG, eggNOG, Swiss-Prot and Pfam. 59,280, 49,345, and 85,922 differentially expressed uniquely assembled transcripts between salt treated and control triticale root samples at three different time points (C12_vs_T12, C24_vs_T24, and C48_vs_T48) were identified, respectively. Expression profile and functional enrichment analysis of DEGs found that some DEGs were significantly enriched in metabolic pathways related to salt tolerance, such as reduction–oxidation pathways, starch and sucrose metabolism. In addition, several transcription factor families that may be associated with salt tolerance were also identified, including AP2/ERF, NAC, bHLH, WRKY and MYB. Furthermore, 14 DEGs were selected to validate the transcriptome profiles via quantitative RT-PCR. In conclusion, these results provide a foundation for further researches on the regulatory mechanism of triticale seedlings adaptation to salt stress in the future.

## Introduction

High salinity stress is one of major abiotic stresses that limits crop production on at least 20% of the cultivated land worldwide^1^. Moreover, this problem has frequently occurred in recent years and became increasingly severe as the rising sea level from global climate warming and inappropriate irrigation practice^1,2^. As a result, the world's available arable land cannot sufficiency meet the demand for food production. Salinity not only inflicts osmotic stress but also ionic stress on crop plants, and secondary stresses such as oxidative stress also often occur along with a consequence of these primary effects^2^. To survive against these stresses efficiently, crop plants have evolved complex mechanisms involving multiple genes and strategies at physiological, molecular and metabolic levels^3^. Therefore, improving and using saline land has great practical value for improving land utilization by adopting biological improvement method^4^. To date, numerous genetic improvement investigations into crop plants have been conducted to elucidate changes in the transcriptome-level of species in response to salt stress, such as Arabidopsis^5^, rice^6^, peanut^7^, sugar beet^8^, cotton^9^, citrus^10^, *Clerodendrum inerme* (L.)^11^, Microalgae *Dunaliella*^12^, kenaf^13^, and wild barley^14^. These studies have revealed the similarity in transcriptome responses among species. However, knowledge of the genetic basis underling triticale involved in salt responses is still nearly unknown. The elucidation of differences in salt tolerance mechanisms in triticale and the genes that play major roles in these mechanisms have been limited by the lack of genomes and transcriptomes information. RNA sequencing, one of the most cost-effective platforms for analyzing gene expression, makes it possible to calculate and analyze genome-wide scale and transcriptome-level of triticale without a reference genome^15–17^.

*Hexapod Triticale* (× *Triticosecale* Wittmack, 1889), the first artificial species created by crossing wheat (*Triticum *spp.) and rye (*Secale cereale* Linnaeus, 1753), is an upland crop and sensitive to salt stress. It not only has a great potential as a grain and forage crop, but also serves as an important black food resource and exhibits significant antioxidant, antihypertensive, and cancer prevention effects^18^. However, there were few studies focusing on identifying important regulatory factors related to salt stress in triticale using bioinformatics methods nowadays as the lack of a reference genome sequence^19–21^. Moreover, these studies only focused on their role in the adaptation process after long-term stress, did not provide a global view of the transcriptome at salt-response stage. Additionally, no experiments were performed to investigate the differently expression genes (DEGs) or transcription factors response to salt stress in triticale. The cultivation of triticale with high salt tolerance is of great practical benefit for the effective use of saline-alkali land to improve grain yield.

In this study, we identified some candidate genes and transcription factors involved in key metabolic pathway that association with salt stress responses in triticale by salt-responsive transcriptome analysis, which will provide a valuable resource for elucidating the molecular mechanisms of triticale salt tolerance.

## Materials and methods

### Plant materials growth and salt stress treatment

The material used in this study was salt-tolerant triticale cultivar TW004. Firstly, the TW004 seeds were surface sterilized in 1% Sodium hypochlorite (NaClO) for 10 min, then washed in distilled water several times, and finally planted in a germination box covered with double-layer filter paper. The germination box was subsequently added with 15 mL distilled water, and placed in an artificial incubator with a controlled temperature (~ 20 °C/25 °C day/light cycle), a 12 h/12 h light/dark cycle. The growth substrate and salt treatments were conducted as previously described^22^. Briefly, TW004 seedlings with consistent growth were selected to plant on a floating plate after 3 days, and placed in a hydroponic container containing a nutrient solution with a controlled temperature (~ 20 °C/25 °C day/light cycle), a 12 h/12 h light/dark cycle. Seven days after incubation, the seedlings were treated using 150 mM NaCl solution. After 0, 12, 24, and 48 h salt stress, the root samples were collected from both control and salt-treated plants with three replications, then immediately frozen in liquid nitrogen and stored at − 80 °C until analysis.

### RNA extraction and cDNA library construction

Total RNA was extracted using TIANGEN column plant RNA extraction kit (TIANGEN, Beijing) and purified with an RNeasy mini kit (QIAGEN, Germantown, MD, USA) following the manufacturer’s instructions. The quality and quantity of total RNA was assessed using a NanoDrop ND1000 spectrophotometer (NanoDrop Technologies, Wilmington, DE, USA), Qubit 2.0 fluorometer (Life Technologies, Carlsbad, CA, USA) and an Agilent 2100 Bioanalyzer (Santa Clara, CA, USA). High-quality RNA was used for cDNA library construction using the Illumina TruSeq Stranded RNA Kit (Illumina, San Diego, CA, USA) according to the manufacturer’s recommendations. The purified cDNA fragments were enriched via PCR for RNA-seq sequencing.

### Transcriptome de novo sequencing

Paired-end sequencing of 21 cDNA libraries were performed using the Illumina HiSeq 4000 platform. The quality of raw reads was checked using FastQC toolkit^23^. The high-quality reads were used to generate a de novo assembly using Trinity^24^. Potential protein-coding genes were identified using TransDecoder-2.0^25^. The completeness of the assembled transcriptome was validated with Benchmarking Universal Single Copy Orthologs (BUSCO 3.0.1)^26^. To further obtain sequence annotation, all uniquely assembled transcripts were mapping to databases nr (NCBI non-redundant protein sequences), GO (Gene Ontology, http://www.geneontology.org/), KEGG (Kyoto Encyclopedia of Genes and Genome, http://www.genome.jp/kegg), eggNOG (evolutionary genealogy of genes: Non-supervised Orthologous Groups), Swiss-Prot, and Pfam using BLAST2GO^27^, respectively.

### Identification of differentially expressed genes and pathway enrichment analysis

Identification of differentially expressed genes (DEGs) were performed using the R package ‘DESeq’^28^. Adjusted *p* values were calculated using the Benjamini and Hochberg method^29^ to control the false discovery rate. The standard for screening DEGs was set as (1) an adjusted *p* < 0.05 and (2) | Log_2_ (a fold change) |≥ 1. The expression patterns of DEGs at different time point were conducted with the STEM^30^. The functional annotation of DEGs was mainly conducted through Gene Ontology (GO) annotation and Kyoto Encyclopedia of Gene and Genome (KEGG) pathway enrichment analysis with agriGO 2.0 (http://bioinfo.cau.edu.cn/agriGO/)^31^ and KOBAS 3.0 (http://kobas.cbi.pku.edu.cn/index.php)^32^, respectively.

### Quantitative RT-PCR (qRT-PCR) validation

The quantitative RT-PCR (qRT-PCR) analysis was conducted using the extracted total RNA of 21 triticale samples subjected to transcriptome analysis in this study. According to the manufacturer’s instruction, RNase-free DNase (Invitrogen, Gaithersburg, MD, USA) was used to purify the total RNA and recombinant M-MLV reverse transcriptase (Invitrogen) was applied to synthesize single-stranded cDNA. qRT-PCR was conducted using gene-specific primers in a 25μL reaction with a 2 × iTaq Universal SYBR Green Supermix (BioRad, Hercules, CA, USA) according to the manufacturer’s instructions. Normalization was done using Actin as an internal control gene as reported in previous studies^33,34^. Primers were designed on different exons to avoid amplification of genomic DNA (Table S1). Relative expression levels were calculated according to the 2^−ΔΔCT^ method^35^.

## Results

### The time-series transcriptome analysis of triticale seedling root

**Figure 1:**
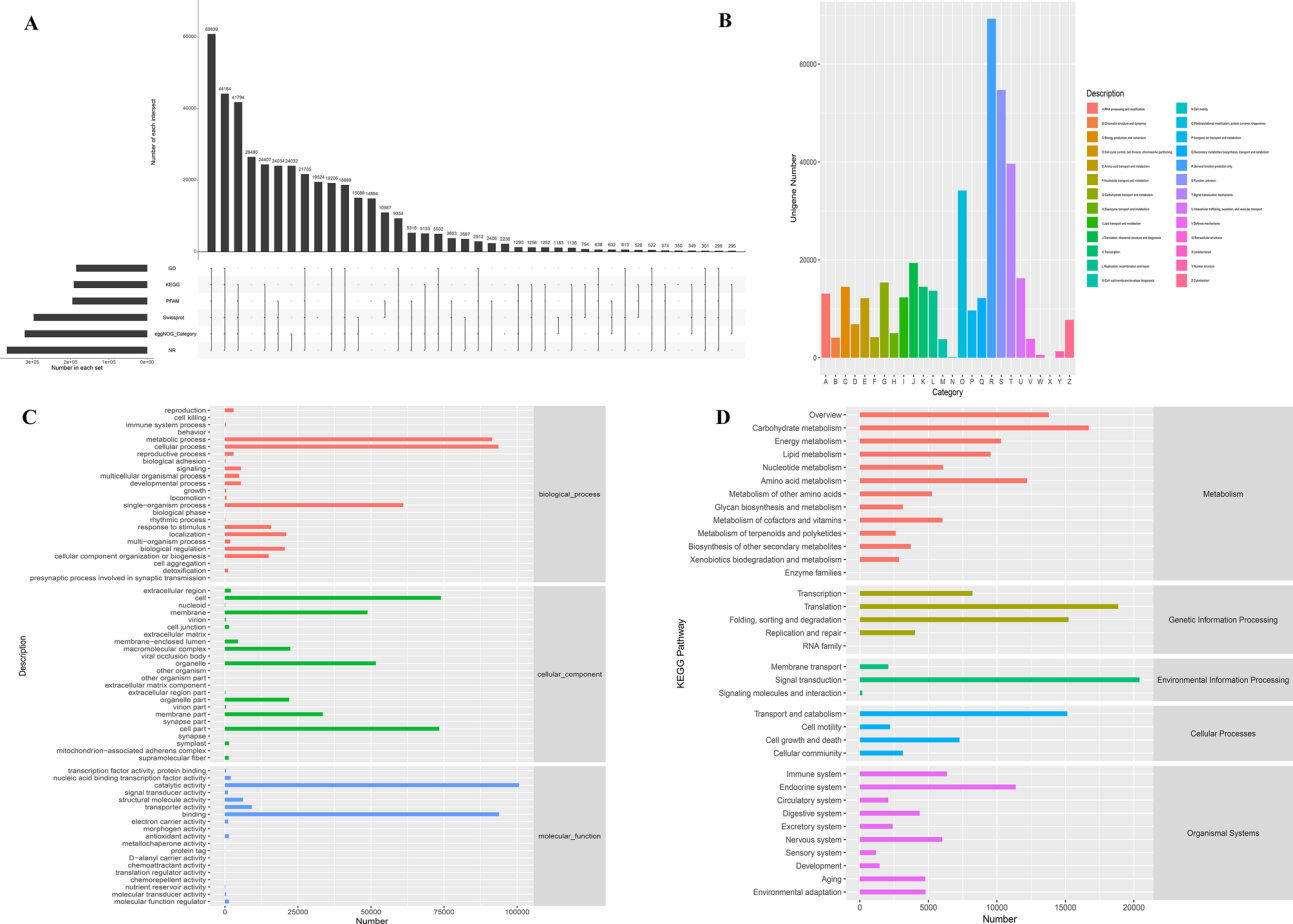
Sequence annotation of all uniquely assembled transcripts generated via de novo assembly in triticale. (**A**) The Upset diagram of statistical results for all uniquely assembled transcripts annotated to databases nr, GO, KEGG, eggNOG, Swiss-Prot, and Pfam. (**B**) All contigs were assigned to database eggNOG and classified into 25 functional categories. (**C**) All contigs were assigned to GO categories and classified into 47 functional terms. (**D**) All contigs were assigned to database KEGG and classified into 33 functional categories.

To obtain a comprehensive understanding of gene expression dynamics in the root of triticale TW004 seedling in response to salt stress, all 21 samples’ cDNA libraries were subjected to de novo transcriptome sequencing (0, 12, 24, and 48 h of controls (C_0h, C_12h, C_24h, and C_48h); and 12, 24, and 48 h of salt-treated plants (T_12h, T_24h, and T_48h)). Total 136.07 Gb clean data were obtained with a Q30 percentage above 91.69% (Table S2), which were further assembled into 877,858 uniquely assembled transcripts (Table S3). Sequence annotation indicated that 41.71% (366,168), 21.05% (184,767), 21.76% (190,980), 22.19% (194,791), 36.39% (319,455), and 33.72% (296,000) of the uniquely assembled transcripts were annotated to the nr, GO, KEGG, Pfam, eggNog, and Swissport databases, respectively. It revealed that most of high-quality uniquely assembled transcripts could significantly match to genes with known functional (Fig. 1A). Among the functional categories, 319,455 (36.39%) contigs were aligned to the eggNOG database and classified into 25 functional categories (Fig. 1B). 184,767 (~ 21%) were classified into different functional terms from three GO categories (Fig. 1C). And 190,980 (~ 21.76%) uniquely assembled transcripts were assigned to 32 KEGG pathways (Fig. 1D). Total 60,839 are simultaneously annotated in all above public databases.

### Identification and annotation of differentially expressed genes

**Figure 2:**
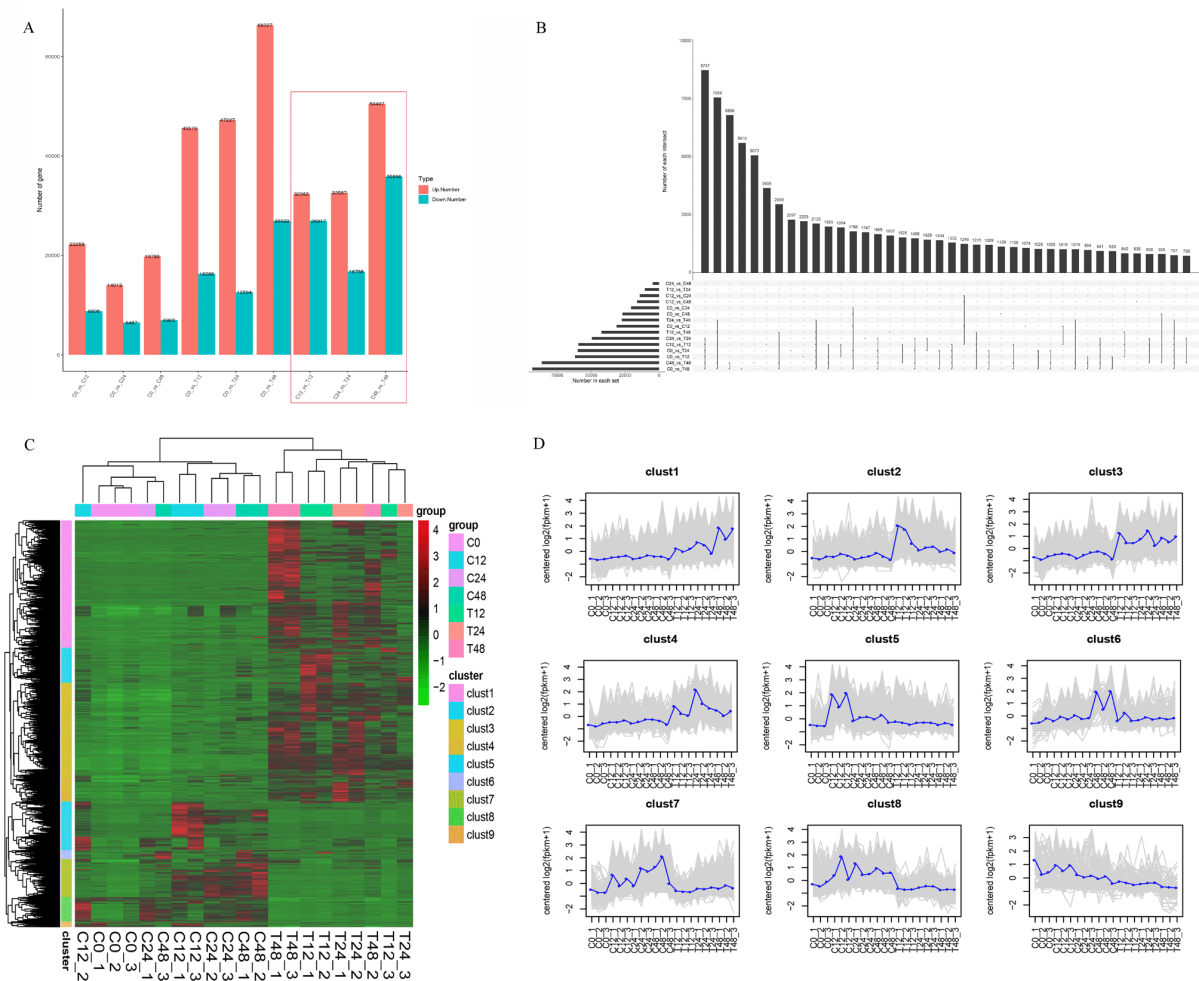
All differentially expressed genes identified in this study. (**A**) Statistical results of all DEGs. (**B**) The numbers of unique and common DEGs among different comparison groups. These DEGs are divided into 9 clusters based on gene expression level and displayed with a heatmap (**C**) and expression tendency (**D**).

**Figure 3:**
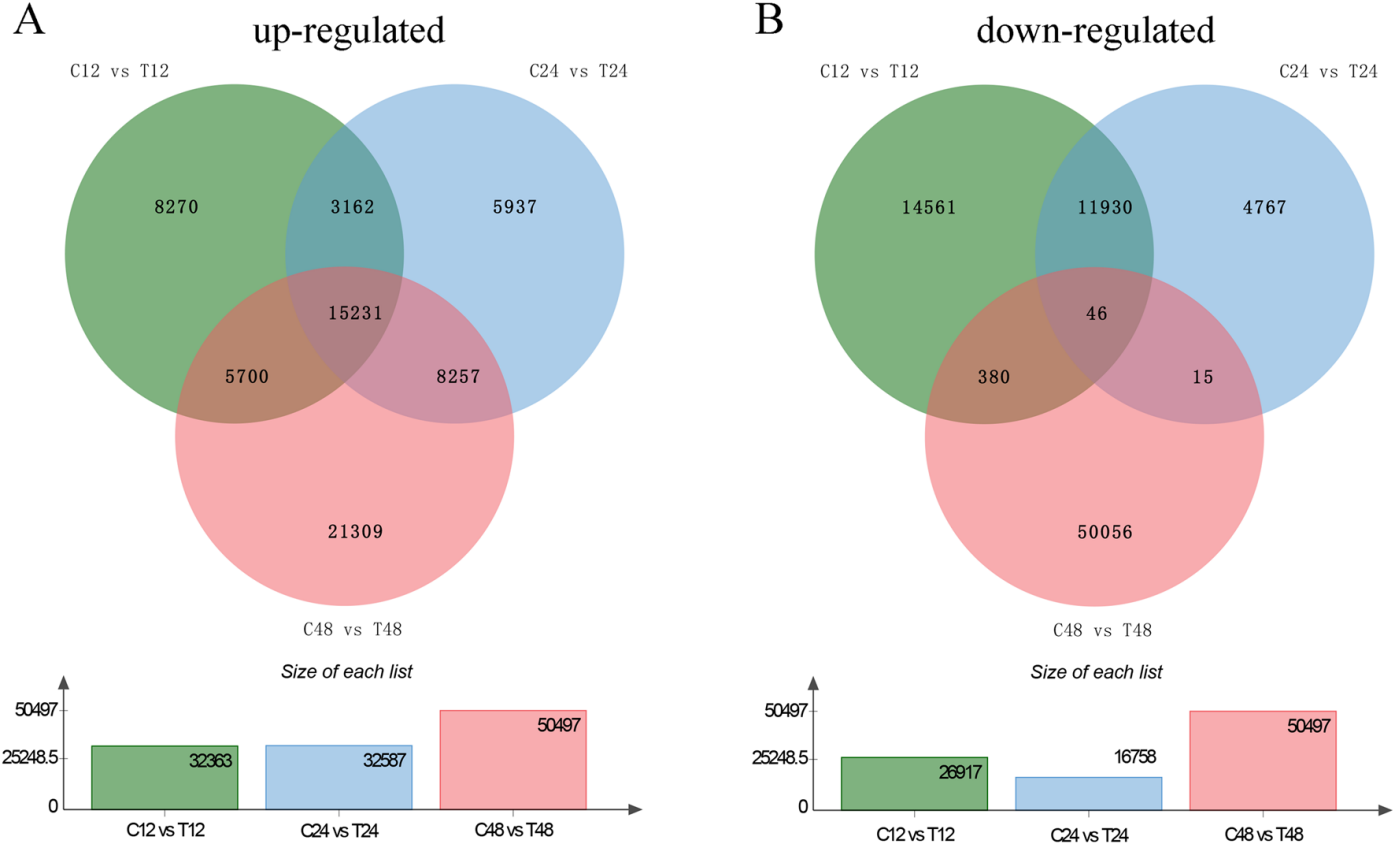
Differentially expressed genes at 12, 24, 48 h after salt stress in triticale. The Venn diagram shows the number of up-regulated DEGs (**A**) and down-regulated DEGs (**B**) after 12 (T_12h), 24 (T_24h), and 48 h (T_48h) of salt stress.

Genes with average expression level (FPKM value) more than one were considered as expressed genes. Overall, about half of uniquely assembled transcripts has been identified as expressed genes (Figure S1). Compared with the 0 h control (C0), the DEGs at 12, 24, and 48 h of salt-free and salt stress triticale were 31,067, 20,500, 26,751, 61,856, 59,821, and 93,249, respectively (Fig. 2A). It means that the genes expression levels can be affected by the circadian clock, so it is necessary to set controls at different time points after salt stress. Finally, 59,280 (32,363 up-regulated and 26,917 down-regulated), 49,345 (32,587 up-regulated and 16,758 down-regulated), and 85,922 (50,497 up-regulated and 35,425 down-regulated) differentially expressed uniquely assembled transcripts were identified between salt treated and control triticale root samples at three different time points (C12_vs_T12, C24_vs_T24, and C48_vs_T48), respectively (Figs. 2A,  3A,B). Certainly, there were also DEGs among the other comparison pairs (Fig. 2B), but they will not be emphatically studied here.

**Figure 4:**
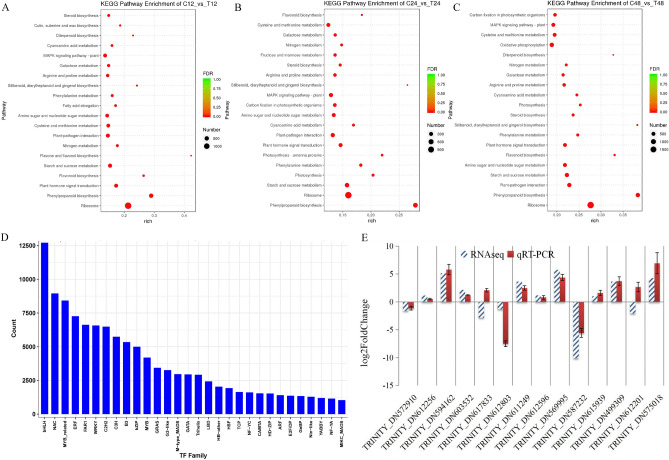
KEGG pathway enrichment analysis of DEGs after 12 (T_12h) (**A**), 24 (T_24h) (**B**), and 48 h (T_48h) (**C**) of salt stress, respectively. (**D**) Transcription factor family identified in triticale. (**E**) Verification of gene expression by real-time quantitative PCR. The black bars represent fold changes based on FPKM calculated from globally normalized RNA-seq data. The red bars with standard errors indicate fold changes based on the relative expression level determined by qPCR using the 2^−ΔΔCT^ method for three biological replicates under saline and normal conditions.

To further provide a global expression profile of uniquely assembled transcripts under salt stress conditions, expression models of all DEGs were created and divided into nine clusters based on a log_2_(fold change) (Fig. 2C,D; Tables S4–S5). The results revealed that the expression patterns of most DEGs were reversed in treatment and control group, that is, most DEGs were up-regulated after salt stress. Of which, the genes in clusters 1–4 were up-regulated under salt stress, while the clusters 6–9 genes were down-regulated, especially after 48 h of salt stress (Fig. 2C,D; Tables S4–S5). GO analysis of these DEGs showed some uniquely assembled transcripts significantly enriched in GO terms of the biological processes related to emergency response to salt stress (Figure S2). For functional categories of DEGs, (1) In molecular function classes, the top five sub-classes are ion binding, phosphatase activity, protein kinase activity, protein serine/threonine kinase activity, and structural molecule activity. (2) In biological process, the top five sub-classes are protein phosphorylation, oxidation–reduction process, carbohydrate metabolic process, response to chemical, and cellular response to chemical stimuli. (3) In cell components, the top five enrichment sub-classes are membrane part, intrinsic component of membrane, integral component of membrane, cell periphery, and plasma membrane (Table S6). Moreover, KEGG pathways enrichment analysis of DEGs identified at 12, 24, and 48 h after salt stress were further conducted and found that DEGs were significantly enriched in specific metabolic pathways in response to salt stress, including phenylpropanoid biosynthesis, starch and sucrose metabolism, oxidative phosphorylation, sugar metabolism, plant hormone signaling transduction, and cysteine and methionine metabolism (Fig. 4A–C). Among them, phenylalanine ammonia-lyase encoding genes TRINITY_DN622507_c1_g2, TRINITY_DN587646_c3_g2, TRINITY_DN571002_c1_g3, TRINITY_DN588048_c0_g4, and tyrosine ammonia-lyase encoding gene TRINITY_DN605273_c2_g1 were enriched in phenylpropanoid biosynthesis, and nine genes, including kinase inhibitor 1 BRI1 encoding gene TRINITY_DN581258_c0_g1 and threonine-protein kinase CTR1 encoding gene TRINITY_DN577259_c2_g1, were enriched in plant hormone signaling pathways.

### Detection of important salt-induced candidate genes and transcription factors

Many biology processes, including signal transduction, cell wall metabolism, defense metabolism, transporters and transcription factors, especially oxidation–reduction system and sugar metabolism pathway, are closely related to salt stress in plant tissues. The transcriptome analysis for salt tolerant triticale identified total 134,990 DEGs at 12, 24, and 48 h after salt stress, and KEGG pathway enrichment analysis found that those candidate DEGs were mainly enriched in plant hormone signal transduction, starch and sucrose metabolism, phenylpropanoid biosynthesis, and flavonoid biosynthesis (Fig. 4A–C). Especially, there were 60 genes well-connected with oxidation–reduction system and sugar metabolism pathway based on results of KEGG pathway enrichment analysis (Table S7). These genes were considered as important salt-induced candidate genes in triticale. Moreover, the expression level of peroxisomal membrane protein encoding genes TRINITY_DN614229_c0_g1 and TRINITY_DN615939_c2_g1 were up-regulated 8.6 times and 5.88 times after salt stress, respectively. Super-oxide dismutase (SOD) encoding genes TRINITY_DN562377_c1_g1 and TRINITY_DN617105_c1_g3 were up-regulated 4.34 times and 5.69 times, respectively. Genes encoding peroxidase (POD), which plays a key role in detoxification of intracellular ROS, were also up-regulated under salt stress.

**Figure 5:**
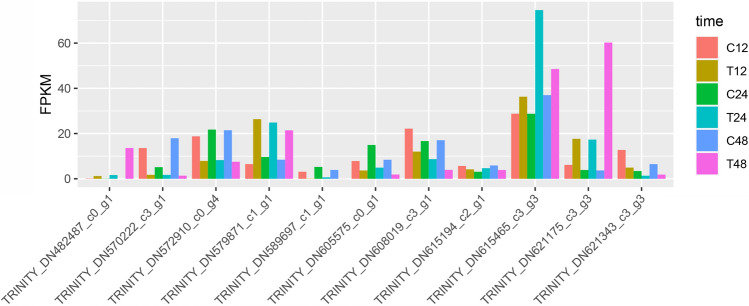
The changes of expression levels of 11 transcription factors under salt stress.

To investigate the transcription factors (TF) in triticale under salt stress conditions, a sequence comparative analysis of all uniquely assembled transcripts of triticale with all public TFs downloaded from PlantTFDB was conducted and ultimately 30 TF families were detected (Fig. 4D). Among them, three transcription factors, bHLH, NAC, and MYB, are the most abundant. Pathway enrichment analysis reveals that members of the transcription factor family, such as bHLH, AP2/EREBP, MYB, WRKY, NAC, MADS, and bZIP, have been identified to participate in the response of plants to salt stress. For example, bHLH including seven up-regulated genes, TRINITY_DN605575_c0_g1, TRINITY_DN608019_c3_g1, TRINITY_DN589697_c1_g1, TRINITY_DN621175_c3_g3, TRINITY_DN572910_c0_g4, TRINITY_DN621343_c3_g3, and TRINITY_DN615465_c3_g3; NAC including four up-regulated genes TRINITY_DN482487_c0_g1, TRINITY_DN615194_c2_g1, TRINITY_DN570222_c3_g1, and TRINITY_DN579871_c1_g1 (Table S7). Transcriptome analysis showed that above transcription factors were up-regulated or down-regulated under salt stress (Fig. 5). The reason for the difference in genes expression levels may be that some metabolic pathways responding to salt stress in triticale were enhanced by the regulation of various physiological and biochemical reactions.

### Validation of DEGs by quantitative RT-PCR

In order to further validate the accuracy of RNA-Seq expression profiling, 14 salt responsive genes were selected for qRT-PCR. The qRT-PCR results were highly consistent with those of RNA sequencing (R^2^ = 0.84, *P* = 0.002) (Fig. 4E). Therefore, the DEGs identified in this study can be considered to have a high accuracy.

## Discussion

Transcriptional regulation plays an important role in the response and adaptation to abiotic stress^36,37^, which could improve the survival rate and biomass yield of plants. Nowadays, many researches about adaptation to abiotic stress have been carried out in plants^38–40^. But, the molecular regulation mechanism of triticale response to salt stress is still unclear. Given the plant roots are highly sensitive to salt and are the primary site of perception to the soil environment, the root samples of triticale TW004 seedlings treated with salt and non-salt were collected for transcriptome sequencing in this study.

The tolerance to salt is closely related with the activity of antioxidant enzymes in plants^41,42^. For example, the accumulation of hydrogen peroxide and hormone concentration significantly changed at 6, 12 and 24 h under salt stress in *Populus tomentosa*^43^. The super-oxide radical and H_2_O_2_ are both reactive oxygen species (ROS). SOD converts superoxide radicals into H_2_O_2_, which is further cleared by POD, CAT or other enzymes^44–46^. The lawn grass root de novo transcriptomes analysis^47^ also found a large number of candidate genes related to the early response to salt stress. In the present study, some candidate genes related to ion transport and antioxidant enzymes were identified, and those genes were enriched in vital metabolic pathways such as ROS scavenger enzymatic system and ion transport in response to NaCl stress. This finding indicated that the time point of significant changes in ion accumulation and antioxidant enzyme activity can be used as an effective marker to indicate significant changes in the expression of salt reactive genes in the short-term adaptation of triticale to salt stress. POD plays a key role in detoxification of intracellular ROS. Glutathione reductase (GR) is an enzyme that catalyzes the oxidized glutathione (GSSG) and NADPH to reduced glutathione (GSH). GSH can reduce hydrogen peroxide (H_2_O_2_) produced in plants. According to Yadav et al*.*^48^, the increase of GSH content can protect plants from oxidative damage and then improve salt resistance. Under salt stress, plant bodies were over-expressed by related enzymes in the glyoxalase synthesis pathway, which increased the activity of various reduced glutathione in transgenic plants and made transgenic tomatoes salt-resistant. Therefore, it is preliminarily speculated that the over expression of genes encoding GR under salt stress might increase the content of GSH, which can enhance the scavenging capacity of reactive oxygen species and reduce the membrane lipid peroxidation, to ultimately reduce the damage caused by salt stress. In this study, the genes encoding sucrose synthase (SuSy) was induced to up-regulate in response to salt stress (Fig. 3A, Table S6). Sucrose is a regulatory factor participated in the stress response for plant adaptation to abiotic stress based on signal transduction^49^. Therefore, it is speculated that the over-expression of SuSy genes regulated the synthesis of sucrose. The genes encoding hexose transporter were up-regulated under salt stress in this study (Table S6), and the hexose transporter can regulate the transport and distribution of sugar in plants^50^. Under abiotic stress, the expression of genes encoding sugar transporter will be inhibited, which in turn affects plant physiological activities^51^. Transcriptional regulation of sugar transporters can change the osmotic pressure of plants in the adverse environment, which may be a response mechanism to salt stress.

Previous studies indicated TFs can help plants resist abiotic stresses by regulating the expression of stress-responsive genes, such as NAC domain-containing protein, ethylene-responsive (ERFs), the AP2 domain/bHLH, MADS-box proteins, zinc finger, WRKY, and MYB^52–62^. Moreover, comparative transcriptome analysis of tolerant and sensitive lines in maize^40^ and rice^63^ also demonstrated that ethylene response-binding proteins play a vital role in enhancing tolerance. In this study, AP2/ERF encoding genes TRINITY_DN567174_c7_g3 and TRINITY_DN582605_c6_g1 were significantly up-regulated after 48 h of salt stress in triticale (Table S7), implying the important roles responses to salt stress through ROS-responsive AP2/ERF transcription factors, as described in Arabidopsis^64^. In *Gossypium hirsutum*^65^ and *Arabidopsis thaliana*^66^, it has been discovered that WRKY is involved in salt tolerance. Seven DEGs encoding WRKY were up-regulated under salinity in this study, suggesting they were involved in the salt tolerance stress response in triticale. Over-expression of bHLH encoding gene IAA-LEUCINE RESISTANT3 (ILR3) could increases salt tolerance in Arabidopsis^67^, which consistent with the result of high salinity induced the up-regulation of bHLH in triticale (Table S7). Moreover, MYB, NAC, bHLH, WRKY and MADS encoding gene were also detected among KEGG^68^ enrichment analysis (Fig. 4A–C), suggesting that multiple TF families may directly involve in responses to salt stress in triticale.

## Conclusions

In this study, triticale TW004 was used to identify candidate genes related to salt stress, and a total of 134,990 DEGs were identified under salt stress. Of them, 30 transcription factors and 60 candidate DEGs were ultimately considered to be involved in salt tolerance responses pathways, such as oxidation–reduction system and sugar metabolism, which will be useful for future functional genomics research under salt stress conditions in crop plants.

## Supplementary information

Supplementary Figure S1.

Supplementary Figure S2.

Supplementary Table S1.

Supplementary Table S2.

Supplementary Table S3.

Supplementary Table S4.

Supplementary Table S5.

Supplementary Table S6.

Supplementary Table S7.

Supplementary Legends.
