# Supplementary figures and images for: Salt-responsive transcriptome analysis of triticale reveals candidate genes involved in the key metabolic pathway in response to salt stress
Source: Sci Rep. 2020 Nov 26;10:20669. doi: 10.1038/s41598-020-77686-8 (PMC7691987; doi:10.1038/s41598-020-77686-8)

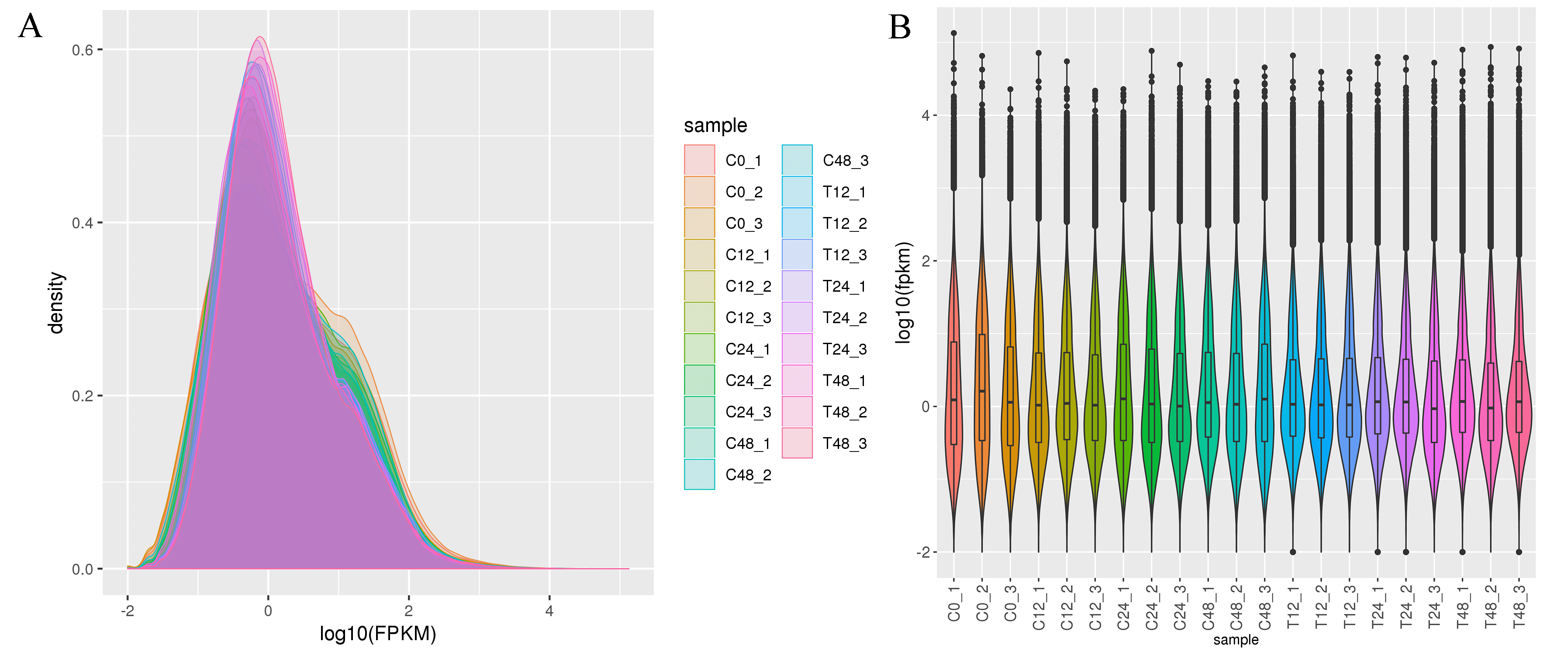

Supplement: Supplementary file 1 — Supplementary Figure S1. [file 41598_2020_77686_MOESM1_ESM.png]
