# Supplementary material for: Salt-responsive transcriptome analysis of triticale reveals candidate genes involved in the key metabolic pathway in response to salt stress
Source: Sci Rep. 2020 Nov 26;10:20669. doi: 10.1038/s41598-020-77686-8 (PMC7691987; doi:10.1038/s41598-020-77686-8)
Supplement: Supplementary file 3 — Supplementary Table S1. [file 41598_2020_77686_MOESM3_ESM.docx]

**Table S1.** Primers used for quantitative RT‐PCR experiments.

| Gene Name | Forward Primer (5′–3′) | Reverse Primer (5′–3′) |
| --- | --- | --- |
| TRINITY_DN572910 | GGTGCTGCAGGACCAAATTG | GGCCTGATTGGTGTCACCAG |
| TRINITY_DN612256 | ATACTCGACGACGGCTTCCG | CGAGTTGCCTGCTGATCGAA |
| TRINITY_DN594162 | TTAACGGCGGCACAGCTAAC | CGCCGTCTTGTCTCCTGTGT |
| TRINITY_DN603532 | TGACAATGCATGCTGCTCGA | TCAGTTGAAGGCGCACTTGC |
| TRINITY_DN617833 | AAAGGATGGCGCAGAATGTG | CACGGCCAGATTAGGCAATG |
| TRINITY_DN612803 | TTCTCGATCTCGCCGTTCTG | CCCAAGACCCTCCTGCTGAT |
| TRINITY_DN611249 | TTCGCCGATCTGAACCACAT | CATCAGCATCTCCCACCAGG |
| TRINITY_DN612596 | CGTTGACGAACCTGCAATCC | ACTTTCGTGAGACGTTCGCG |
| TRINITY_DN569995 | ATCCGCATTGACTTCCATCG | TGCCTCTGATCCTAGCCCAC |
| TRINITY_DN615939 | TCGGATGAAGAACACAGGCA | TGGAAGCCCTGAGACCTTGA |
| TRINITY_DN499309 | TCAGTCATTGGCCGTGCATT | GCTAAACGGCCACCAGCATT |
| TRINITY_DN612201 | CGCGACGAGAACCTGAAGTC | TCCCTTTGAGAGTCCCCCAC |
| TRINITY_DN575018 | CCCCGGAGGTCCTCTCTAGA | CAAAAGGATATGCCCCGACA |
| TRINITY_DN587232 | AGTCGATCTCGTCGTGCTCC | GCCACTTCAGCATGCACATC |
