# Supplementary material for: Salt-responsive transcriptome analysis of triticale reveals candidate genes involved in the key metabolic pathway in response to salt stress
Source: Sci Rep. 2020 Nov 26;10:20669. doi: 10.1038/s41598-020-77686-8 (PMC7691987; doi:10.1038/s41598-020-77686-8)
Supplement: Supplementary file 5 — Supplementary Table S3. [file 41598_2020_77686_MOESM5_ESM.docx]

**Table S3.** Total number of uniquely assembled transcripts in triticale.

|  | Contig | Transcript | Unigene |
| --- | --- | --- | --- |
| Sequence Number | 3013177 | 1575852 | 877858 |
| Max. Length (bp) | 24877 | 16003 | 16003 |
| Mean Length (bp) | 268 | 736 | 605 |
| N50 (bp) | 315 | 1104 | 981 |
| N50 Sequence No. | 512356 | 307584 | 163602 |
| GC (%) | 48.47 | 49.13 | 48.92 |
