# Supplementary material for: Salt-responsive transcriptome analysis of triticale reveals candidate genes involved in the key metabolic pathway in response to salt stress
Source: Sci Rep. 2020 Nov 26;10:20669. doi: 10.1038/s41598-020-77686-8 (PMC7691987; doi:10.1038/s41598-020-77686-8)
Supplement: Supplementary file 10 — Supplementary Legends. [file 41598_2020_77686_MOESM10_ESM.docx]

**Supplementary Materials**

**Additional file 1: Table S1.** Primers used for quantitative RT‐PCR experiments.

**Additional file 2: Table S2.** The characteristics and total number of reads for each sample.

**Additional file 3: Table S3.** Total number of uniquely assembled transcripts in triticale.

**Additional file 4: Table S4.** Standardization of gene expression levels for DEGs.

**Additional file 5: Table S5.** DEGs divided into 9 clusters based on their expression tendency.

**Additional file 6: Table S6.** GO enrichment analysis results of DEGs.

**Additional file 7: Table S7.** Candidate genes and transcription factors related to salt stress in triticale.

**Additional file 8: Figure S1.** The density distribution of uniquely assembled transcripts FPKM value.

**Additional file 9: Figure S2.** GO enrichment analysis of DEGs after 12 (T_12h) (**A**), 24 (T_24h) (**B**), and 48 hours (T_48h) (**C**) of salt stress, respectively.
